# Zeolites and Zeolite-Based Materials at the Biointerface: From Haemostasis and Biomolecule Separation to Theranostic Applications

**DOI:** 10.3390/molecules31183183

**Published:** 2026-09-10

**Authors:** Olimpia Tammaro

**Affiliations:** Department of Applied Science and Technology, Politecnico di Torino, Corso Duca degli Abruzzi 24, 10129 Turin, Italy; olimpia.tammaro@polito.it or oli.tammaro@gmail.com

**Keywords:** nanozeolites, theranostics, external surface, surface chemistry

## Abstract

Zeolites are crystalline microporous aluminosilicates whose tunable porosity, ion-exchange capacity, surface charge, and chemical robustness make them versatile materials at the biointerface. This review surveys three converging domains of zeolite biomedicine. First, haemostasis and wound healing, where water adsorption and Ca^2+^ release drive procoagulant activity, from the QuikClot generation to strategies that mitigate the exothermic response and to flexible zeolite–textile dressings. Second, the separation, immobilization, and sensing of biomolecules, where external surface area, hierarchical porosity, and surface chemistry—rather than intracrystalline sieving alone—govern the interaction with proteins and nucleic acids in complex matrices. Third, the emerging design of zeolite-based theranostic platforms integrating drug delivery, imaging, and stimuli-responsive therapy, enabled by the transition from bulk crystals to surface-engineered nanozeolites. Across all three domains, a single lesson recurs: the biological behaviour of zeolites is governed by the external surface rather than by molecular sieving, and the chemical integrity of the framework under working conditions is a design parameter that is reported only sporadically. We further show that the theranostic literature reaching in vivo validation is dominated by zeolite-like imidazolate frameworks, whereas the evidence for aluminosilicate zeolites remains largely in vitro—the gap that most urgently needs closing. The successes of ZIFs should therefore be read as structural inspiration for zeolite design rather than as direct evidence for aluminosilicate clinical translation.

## 1. Introduction

**Figure 1:**
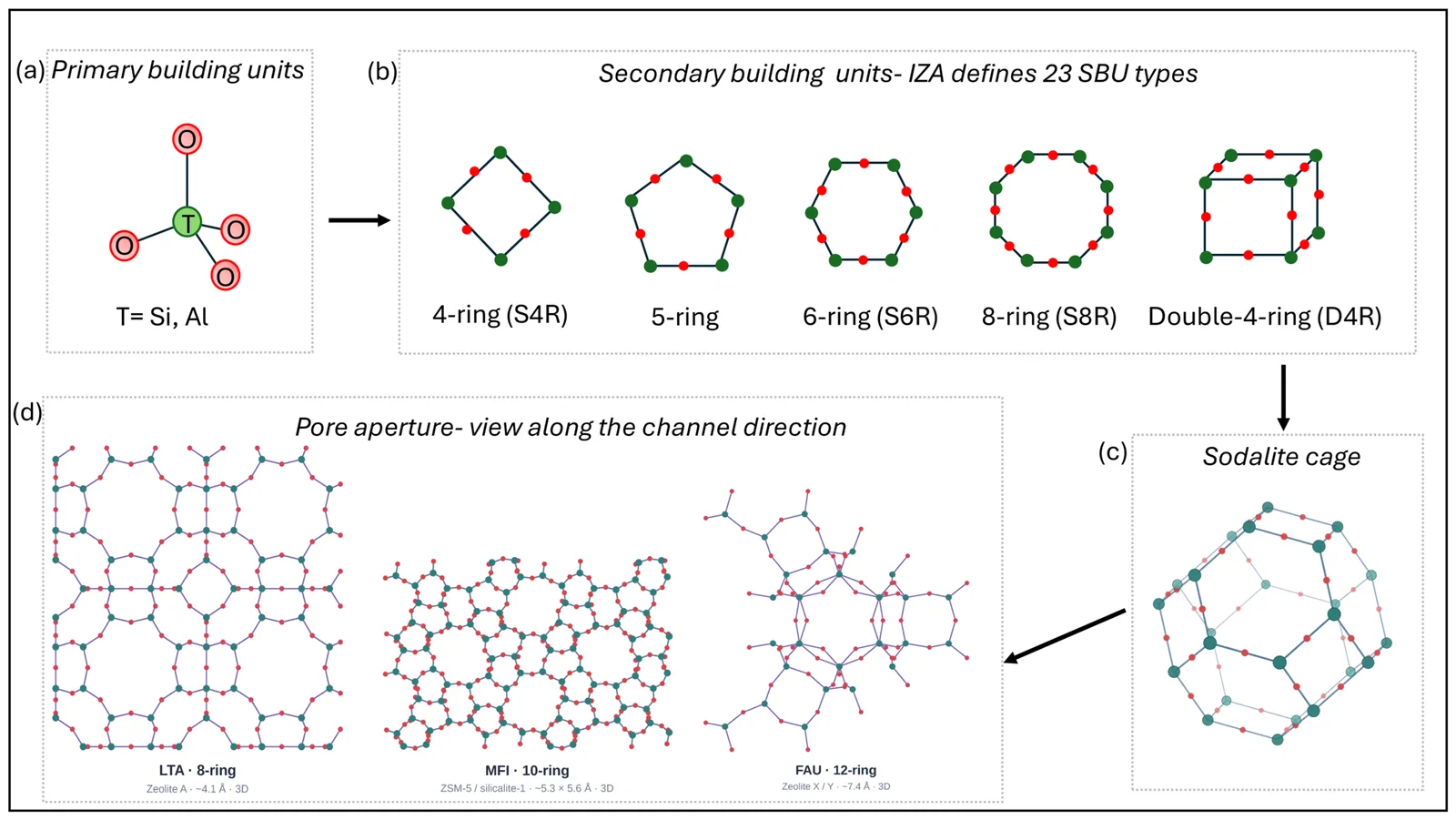
From building units to pore apertures in zeolite frameworks. (**a**) The primary building unit is the TO_4_ tetrahedron, where T is Si or Al. (**b**) Corner-sharing of bridging oxygen atoms generates the secondary building units (SBUs); the Structure Commission of the International Zeolite Association (IZA) currently recognizes 23 SBU types, occurring as single rings (4-, 5-, 6- and 8-membered), double rings such as the double 4-ring (D4R), and more branched arrangements. (**c**) SBUs assemble into polyhedral cages such as the sodalite cage (β-cage), whose mode of connection determines the resulting topology. (**d**) Pore apertures of representative frameworks, shown as projections along the channel direction and generated from IZA crystallographic data: LTA (8-ring, ~4.1 Å), MFI (10-ring, ~5.3 × 5.6 Å) and FAU (12-ring, ~7.4 Å), illustrating the progression from small- to large-pore frameworks. T atoms are shown in green and bridging oxygen atoms in red.

Zeolites are inorganic microporous tectosilicates, of natural or synthetic origin, characterized by a well-defined framework that yields a porous crystalline solid. Isomorphous substitution of the tetrahedral T atoms generates the wider family of zeotypes: aluminophosphates (AIPO), silicoaluminophosphates (SAPO), and titanosilicates such as TS-1. Heteroatoms such as B, P, Sn, Ti, Fe, Ge, Ta, and V can all be introduced into the framework. This chemical versatility is what allows acidity, redox behaviour, and hydrophilic/hydrophobic character to be tuned [1]. The zeolitic imidazolate frameworks (ZIFs) discussed later in this review are not zeolites at all but a subclass of metal–organic frameworks (MOFs) built from metal ions and organic imidazolate linkers; they adopt zeolite-like topologies but are chemically unrelated to aluminosilicates, and the term “zeolite-like” refers only to this topological analogy [2,3]. The structure, shared by all members of the family, consists of a tetrahedral arrangement of tetravalent silicon cations (Si^4+^) and trivalent aluminum cations (Al^3+^), each surrounded by four oxygen anions (O^2−^). The TO_4/2_ tetrahedra, where T represents either Si or Al, form the primary building unit (Figure 1); by sharing corner oxygen atoms, these units combine into more complex structures through secondary building units [4]. A representative example is the sodalite cage (β-cage), a truncated-octahedron unit that, depending on how the cages are connected, generates different framework topologies: linking sodalite cages through double 6-rings yields the faujasite (FAU) framework, whereas connecting them through double 4-rings yields the Linde Type A (LTA) (Figure 1).

By exploiting the bridging oxygen atoms, zeolites can adopt various spatial configurations (single rings, double rings, polyhedral units, or more intricate geometries), generating a framework with a regular distribution of molecular-sized pores (channels) and cavities (cages), typically ranging from about 3 to 12 Å, which underlies their molecular-sieving behaviour [5,6]. The SiO_4_ tetrahedra are electrically neutral, whereas the AlO_4_ tetrahedra carry a negative charge; this intrinsic charge imbalance is compensated by extra-framework cations (such as Na^+^, K^+^, Ca^2+^, Mg^2+^, NH_4_^+^) generally named as counter-cations [7,8]. The general formula for these tectosilicates is:

Me_x/z_[Al_x_Si_(1−x)_O_2_]·wH_2_O

where Me is the cation, z its valence, and x ≤ ½, an upper limit imposed by Löwenstein’s rule, which forbids Al–O–Al linkages and therefore constrains the framework to Si/Al ≥ 1 [9,10]; computational studies have nevertheless predicted violations of this rule in some protonated frameworks [11]. The value of w varies with the type of zeolite, the Si/Al ratio, the cation composition, temperature, and the partial pressure of water vapour in the surrounding environment.

Zeolites are classified as synthetic when produced artificially in the laboratory, or natural when they occur as minerals. The term “zeolite” was first coined in 1756 by the Swedish mineralogist Axel Fredrik Cronstedt, who discovered the mineral stilbite: on heating it, he observed steam released from water adsorbed within its structure, and named it from the Greek *zein* (to boil) and *lithos* (stone) [12]. To date, more than 250 framework types have been approved, about 40 of which also occur as natural minerals [13]; each new framework, whether synthesized or natural, is approved by the IZA Structure Commission (IZA-SC) and identified with a three-letter code. It should be highlighted that such a code designates a topology rather than a specific material: a single framework type may correspond to many compositions, so that zeolites X, Y, and ultrastable Y all share the FAU topology while differing in Si/Al ratio, extra-framework cations and, consequently, in their behaviour at the biointerface. The same topology may moreover occur in both synthetic and natural form: the MFI framework of the synthetic zeolite ZSM-5 is also found as the mineral mutinaite [14,15].

Natural zeolites typically form in alkaline environments through the alteration of volcanic sediments, occurring as minerals such as stilbite, clinoptilolite, erionite, analcime and mordenite. However, they can be relatively expensive and show structural imperfections and compositional variability depending on their source, which limits their uniformity for specific applications. A further consideration, essential in a biomedical context, is that not all natural zeolites are benign: fibrous erionite is classified by the International Agency for Research on Cancer as a Group 1 human carcinogen and is more potent than asbestos in inducing malignant mesothelioma, environmental exposure in Cappadocian villages having caused mesothelioma mortality of epidemic proportions [16]. Fibre morphology, aspect ratio, and biopersistence—rather than chemical composition alone—therefore govern the safety of a zeolite, a caveat that applies to any biomedical use of natural zeolites. Laboratory synthesis, developed from the pioneering hydrothermal work of the 1940s, was later transformed by the introduction of organic structure-directing agents. These agents are bulkier than alkaline inorganic cations and therefore require fewer framework aluminum atoms, giving access to high-silica frameworks with greater hydrothermal stability, stronger acidity, and tunable hydrophobicity that purely inorganic systems cannot reach [1]. Synthesis has therefore been instrumental in expanding their commercial use [17,18]. The most widely used frameworks are FAU, Linde Type A (LTA) and MFI, with the faujasite framework particularly valued for its high stability and large pore size [19,20]. The framework types referred to throughout this review, together with their pore apertures and the sections in which they appear, are collected in Table 1.

This review is organized around three application areas in which zeolites act directly at a biological interface: haemostasis and wound healing, the separation, immobilization and sensing of biomolecules, and theranostic platforms. Applications in which the zeolite is not in contact with a biological interface, or which have been reviewed in detail elsewhere, fall outside its scope; these include silver-exchanged antibacterial zeolites for dental and orthopedic devices [37,38], zeolites as scaffolds in bone tissue engineering, and the oral use of clinoptilolite as a dietary supplement [36]. It is worth noting that the only microporous silicate so far approved as a drug rather than as a device is sodium zirconium cyclosilicate [39], used to treat hyperkalaemia, which shows that regulatory translation of this class of materials is possible but so far exceptional.

One application area deserves explicit acknowledgement even though it lies outside the biointerface focus adopted here: bone tissue engineering. Zeolites are attractive scaffold components because their porosity and biocompatibility support cell adhesion and proliferation, and because ion exchange lets them act as reservoirs of bioactive cations—most notably strontium, which promotes osteoblast differentiation while suppressing osteoclast-mediated resorption. Reported strategies include zeolite–biopolymer composite scaffolds that combine framework bioactivity with a compliant matrix, and Sr^2+^-exchanged zeolite A coatings on 3D-printed titanium implants, which released strontium and promoted new bone formation both around and within the implant in vivo [40,41]. Because these systems operate as solid implants or coatings rather than at a fluid/tissue biointerface, and because the field now has its own dedicated reviews, we do not treat it in depth here and refer the interested reader to those specialized surveys [42].

The characteristic crystalline structure gives these materials a set of properties that underpin their applications, most of which are governed by a single compositional variable, the Si/Al ratio: a low ratio implies a highly charged, hydrophilic framework with many exchangeable cations, whereas progressive dealumination yields more hydrophobic, thermally and chemically more robust materials with fewer ion-exchange sites. The bridging Si–OH–Al hydroxyls associated with framework aluminum also act as Brønsted acid sites, while Lewis acid sites appear where aluminium becomes coordinatively unsaturated once the framework is damaged, for instance by steaming [15]. The features most relevant to biomedicine are summarized below.

- Porosity and surface area. Zeolites combine a high surface area with well-defined pore sizes, typically between 0.3 and 1.2 nm, which accommodate guest molecules and enable applications in catalysis and adsorption [5,6].
- Ion-exchange capacity. Zeolites can exchange their extra-framework cations without altering the framework. In biomedicine, this allows them to sequester toxic ions—the basis of their established use in removing heavy metals and ammonium from water—or release therapeutic ions such as calcium or magnesium [2,8,43].
- Thermal and chemical stability. Zeolites are thermally stable and, in neutral to mildly acidic media, resistant to chemical degradation, making them suitable for a range of environments, including biological systems [20]. This robustness is not unconditional: it increases with the Si/Al ratio, and both aluminum-rich and highly siliceous frameworks are susceptible to dissolution and dealumination under alkaline conditions, a limitation that becomes practically relevant during protein elution and regeneration cycles (Section 2.2) [2].
- Biocompatibility. Certain zeolites, especially sodium- and potassium-rich ones, show good biocompatibility and do not provoke a significant immune response, which is essential for in vivo use; the safety of clinoptilolite-based materials in vivo has been reviewed in detail [36]. Biocompatibility is, however, a property of the individual material rather than of the class as a whole: it depends on chemical composition, particle size and dose and, above all, on fibre morphology, as the carcinogenicity of fibrous erionite noted above illustrates [2].
- Accessible external surface and tunable surface chemistry. Although the micropores are too narrow to admit most biomacromolecules, the external surface can be functionalized with organic linkers, polymers, targeting ligands, or imaging labels, while its charge and hydrophilic/hydrophobic balance are tuned through the Si/Al ratio and the extra-framework cations. This surface, rather than the intracrystalline void, mediates most of the interactions discussed in the following sections [2].

## 2. Biomedical Application of Zeolites

In the following sections, we examine some of the most representative biomedical applications of zeolites. For each, we briefly outline the clinical problem and its relationship to the specific properties of these materials.

### 2.1. Haemostasis and Wound Healing

One biomedical application in which the properties of zeolites have been extensively studied is haemostasis, which mainly exploits their adsorption capacity and intrinsic negative surface charge.

The term haemostasis, coined by the ancient Greeks from *haíma* (blood) and *stasis* (stoppage), describes the physiological process in which clot formation at a specific site stops bleeding. Uncontrolled, excessive bleeding can progress to haemorrhagic shock and death. This may occur not only in battlefield trauma but also in domestic accidents, surgery, and disorders associated with diabetes, congenital disease, or drugs; an open wound is also susceptible to infection and further complications. Although the need to stop bleeding has been recognized since antiquity, it remains of fundamental clinical importance today, especially in emergency and surgical settings [44,45].

**Figure 2:**
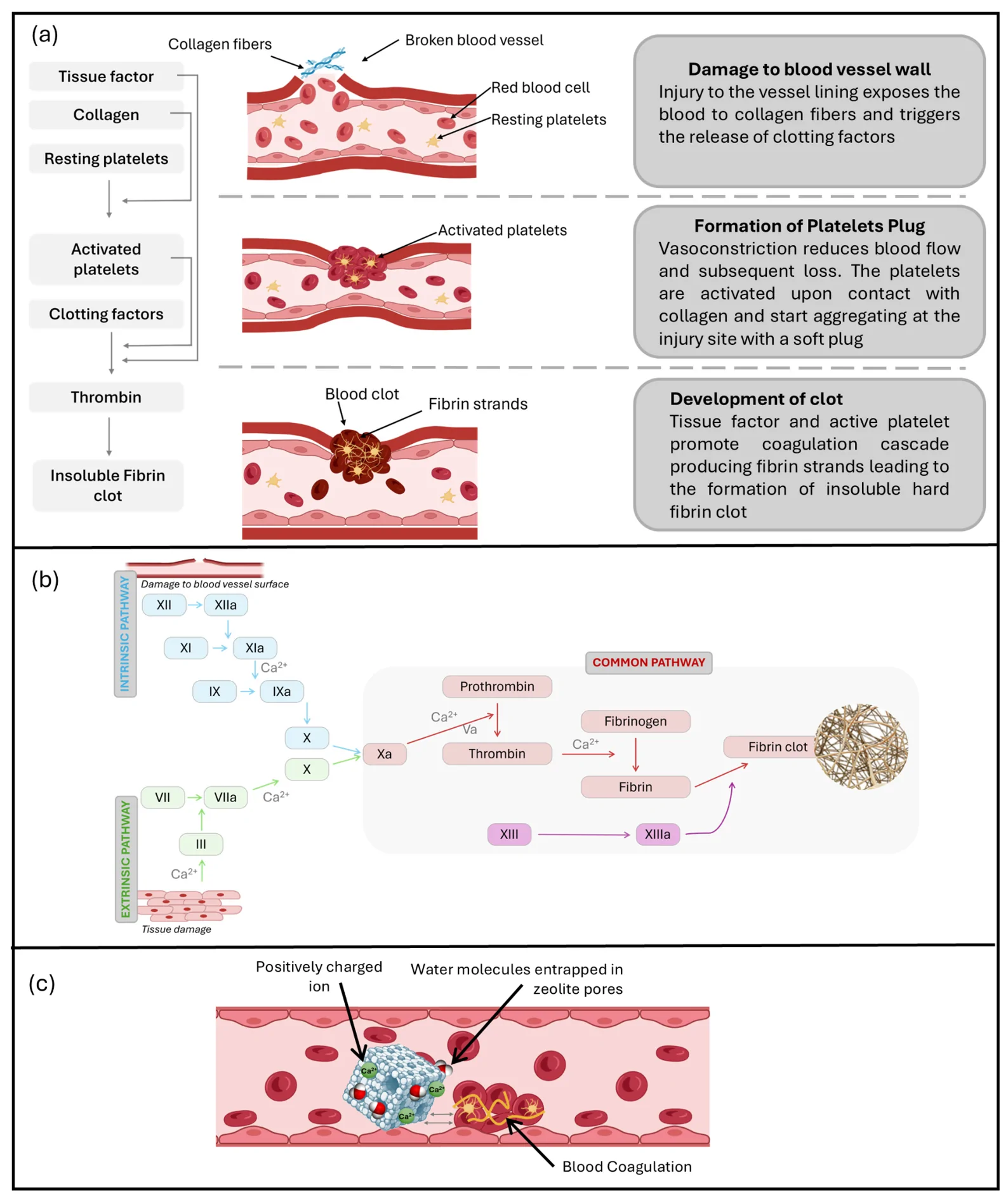
Schematic of blood-clot formation in a broken vessel (**a**). The coagulation cascade: the active intrinsic and extrinsic pathways converging on fibrin-clot formation (**b**). Schematic of the influence of zeolites on blood coagulation, exploiting the water molecules and Ca^2+^ entrapped in the pores to concentrate blood cells and clotting factors (**c**).

Haemostasis can be summarized in three coordinated mechanisms [46]: (i) vasoconstriction, (ii) formation of a platelet plug, and (iii) blood clotting. Vasoconstriction compresses the vessels, reducing blood flow and increasing the exposure of blood to collagen fibres in the vessel wall. Platelets are activated on contact with collagen and release mediators that further promote vasoconstriction and aggregation at the injury site. This initial soft plug is then converted into a more stable one through the coagulation cascade, driven by the concurrent presence of plasma proteins, coagulation factors, calcium ions (Ca^2+^), and activated platelets (Figure 2).

The coagulation cascade comprises two pathways: the intrinsic (contact-activation) and the extrinsic (tissue-factor) pathway. In both, the final step involves the activation of factor X and the presence of factor Va [47].

A critical determinant of this phase is the availability of calcium ions (Figure 2b). Activated factor X (factor Xa) catalyzes the conversion of prothrombin to thrombin in the presence of Ca^2+^, while factor Va acts as a cofactor assisting formation of the prothrombinase complex [48]. Thrombin then reinforces the plug through two mechanisms: (i) conversion of soluble fibrinogen into insoluble fibrin and (ii) activation of factor XIII (XIIIa), which cross-links fibrin.

Materials that accelerate one or more of these processes are termed haemostatics [48]. Their efficacy defines three classes: (i) factor concentrators, which absorb water from blood and thereby concentrate blood components at the hemorrhage site; (ii) procoagulants, which activate the coagulation cascade; and (iii) mucoadhesive agents, which form a physical barrier to blood flow.

In this context, zeolites act through multiple mechanisms. Their well-defined porous structure, with large cage-like cavities, traps substantial volumes of water together with positively charged ions, typically Ca^2+^ and Na^+^, allowing them to concentrate coagulation factors and platelets within hemorrhaging blood as factor concentrators.

By exploiting their ion-exchange capacity, zeolites can also act as procoagulants: the release of Ca^2+^ entrapped in the pores accelerates the intrinsic coagulation pathway and enhances fibrin production, favouring a stable plug (Figure 2c). Li and colleagues demonstrated in vitro the efficient release of Ca^2+^ from zeolite upon exposure to blood or saline through a cation-exchange mechanism [21]: introducing zeolite into blood or NaCl solution releases the Ca^2+^ held in the zeolite, which is replaced by Na^+^ and K^+^, accounting for the observed rise in Ca^2+^ and the corresponding decrease in Na^+^ and K^+^. Measuring prothrombin time (PT) and activated partial thromboplastin time (APTT)—the primary clinical indicators of the extrinsic and intrinsic pathways, respectively [47]—they observed a concentration-dependent reduction in APTT with no significant change in PT, indicating that zeolite accelerates the intrinsic pathway and shortens clotting time.

Importantly, water adsorption in zeolites is exothermic and can raise the material’s temperature to about 60 °C, potentially causing tissue damage that offsets the benefit. One mitigation strategy is to modify the chemical composition or the physical form. Ahuja and co-workers investigated such modifications in a swine model of battlefield injury [49]: the granular Linde 5A zeolite was chemically modified by cation exchange, replacing calcium with sodium, barium, or silver, while physical modification consisted of packaging the zeolite in a fabric bag and/or incorporating chitosan. Ionic substitution reduced the in vivo local surface temperature peak by about 10 °C (relative to ~60 °C at the surface for untreated zeolite [50]) without causing necrosis. Physical modification did not lower the temperature, but the bagged zeolite produced no histological changes in the exposed arteries, suggesting that a physical barrier between zeolite and tissue can be protective.

A zeolite compositionally similar to Ahuja’s is the commercial QuikClot granular powder (QC, Z-Medica), cleared by the FDA through the 510(k) pathway for external use in 2002 [51], consisting of granular zeolite with ~1% residual moisture. QC is biologically inert and sterile and acts by rapidly absorbing water from blood; its efficacy in humans has been supported by numerous studies, particularly in U.S. military use, with roughly 100% efficacy as a primary intervention and 92% in controlling bleeding [52]. These benefits came with drawbacks: the exothermic entrapment of water raised local temperatures to ~95 °C at the surface and ~50 °C in the underlying tissue [53], causing thermal injury and necrosis that complicate healing. In 2008, the product was withdrawn from military use because the granules were difficult to remove—with possible inflammatory granulomas or abscesses [54]—and were displaced under high-pressure bleeding [55].

The original product was replaced by two alternatives of similar composition, both cleared by the FDA through the 510(k) pathway for external use in 2006: Advanced Clotting Sponge (ACS) and ACS Plus (ACS+). ACS consists of zeolite beads encased in gauze, easing handling and removal, especially in irregular cavities [56]. ACS+ uses similarly packaged synthetic zeolite beads that are pre-hydrated to limit the thermal increase associated with QC and ACS [57]. A further strategy is to combine the zeolite with a second mineral such as bentonite: the commercial CoolClot, with a bentonite-to-zeolite weight ratio of 2:1, reached a maximum of ~40 °C in vivo in rats [58].

**Figure 3:**
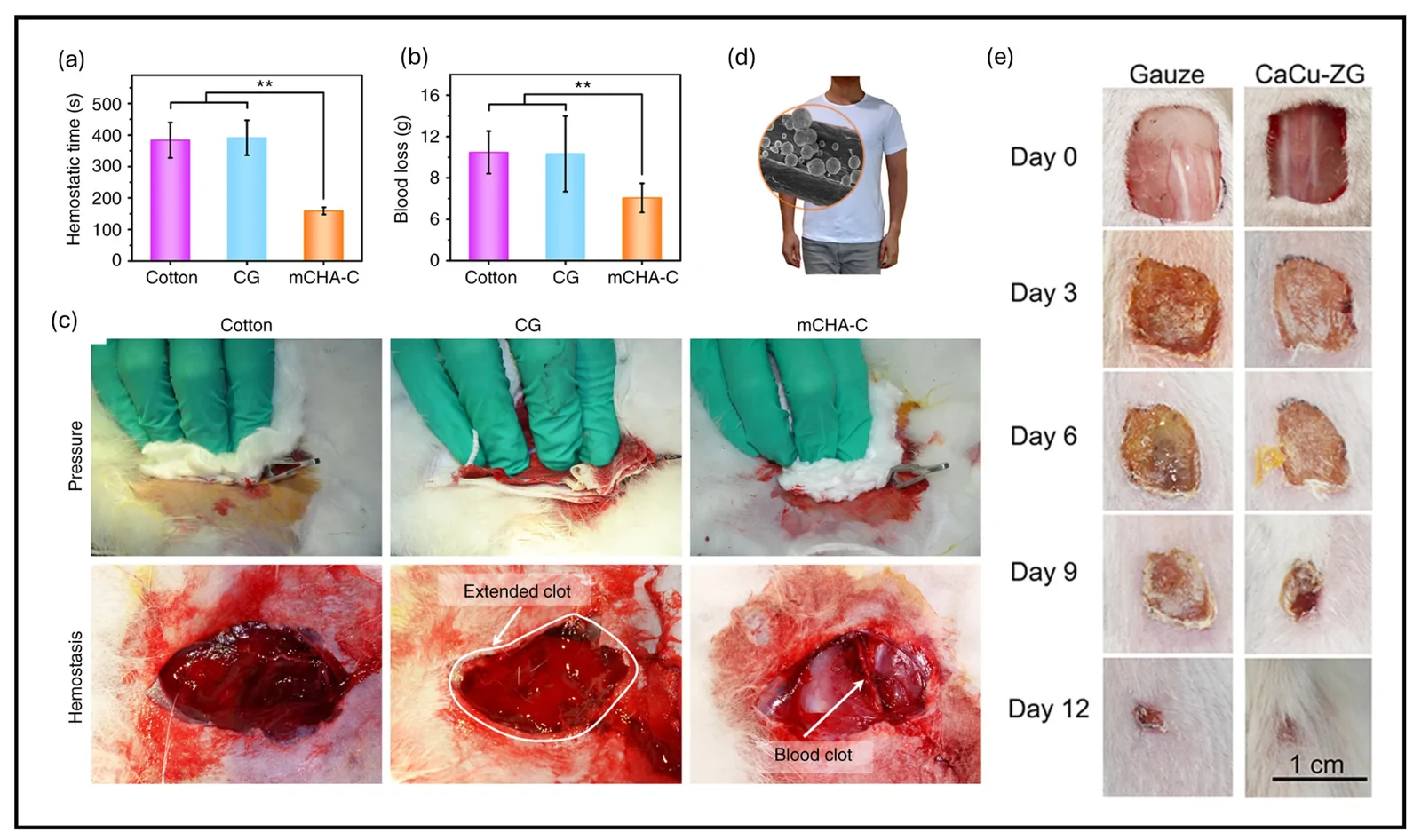
In vivo haemostatic performance of zeolite-based gauzes. (**a**,**b**) Quantitative analysis of haemostasis time (**a**) and blood loss (**b**) in a rabbit femoral artery injury model (mean ± SD, *n* = 8; error bars indicate SD). (**c**) Representative images of haemostasis under manual compression in a lethal rabbit femoral artery injury model treated with cotton, Combat Gauze (CG), or mesoporous chabazite–cotton (mCHA-C). (**d**) Representative image of the mCHA/T-shirt haemostatic material. (**e**) Photographs of wound areas on successive days after treatment with medical gauze or calcium–copper zeolite gauze (CaCu-ZG, 1.22‰ Cu). Panels (**a**–**d**) adapted from [31]; panel (**e**) adapted from [62].

A more recent strategy addresses two long-standing limitations of granular haemostats—component leaching and difficult removal—by anchoring the zeolite onto a flexible textile support. Yu and co-workers grew mesoporous single-crystal chabazite in situ on cotton fibres (CG) through a template-free route, obtaining a tightly bonded, flexible zeolite–cotton hybrid dressing [31]. The chabazite particles remained firmly attached, with less than 1% leaching after 10 min of sonication, and the hybrid retained high procoagulant activity even after exposure to water flow. It outperformed most clay- or zeolite-based inorganic haemostats in procoagulant activity, loss of active components, and scalability (Figure 3a–d). This work marks a shift from loose granular powders toward robust, wearable haemostatic devices.

The same design philosophy—firmly immobilizing the active zeolite within a flexible, removable matrix—underpins several recent composite haemostats. Zhu and co-workers grew zeolite in situ within a poly(vinyl alcohol) sponge by hydrothermal synthesis, obtaining a zeolite–PVA sponge that achieved rapid haemostasis in a lethal massive-hemorrhage model [59]. Following a similar rationale, Jin and co-workers embedded zeolite particles in a regenerated-cellulose aerogel (Z-RCA), in which cellulose nanofibres entangle the crystals through hydrogen bonding: this firm anchoring markedly reduced zeolite detachment—one of the causes of distal thrombosis—while limiting exothermic tissue damage and preserving efficient haemostasis [60]. Composite mineral haemostats have also been pursued: Wang and co-workers combined kaolin and zeolite in a single dressing and demonstrated a synergistic procoagulant mechanism, with each component reinforcing distinct steps of clot formation [61].

Beyond bleeding control, functionalized zeolite dressings increasingly target the wound environment itself. By exchanging both calcium and copper into a zeolite gauze, Wang and co-workers obtained a calcium–copper zeolite gauze (CaCu-ZG) that combined procoagulant activity with antibacterial action and good biocompatibility. The dressing shortened the clotting time relative to medical gauze (from ~509 to ~282 s) and promoted healing in an infected-wound model, and the copper exchange did not compromise the procoagulant performance (Figure 3e) [62]. In a related, prehospital-oriented design, Edwards and co-workers incorporated zeolite Y into a greige-cotton dressing together with silver nanoparticles and cellulose-crosslinked ascorbic acid, obtaining a haemostatic gauze with antibacterial activity for prolonged field care. The ammonium-Y-treated fabric initiated fibrin formation comparable to a standard hemorrhage-control dressing, while the silver and ascorbic-acid finishes conferred antibacterial protection [63].

The performance of such devices must ultimately be confirmed under realistic conditions. Jia and co-workers compared a bio-zeolite gauze with QuikClot Combat Gauze on major bleeding in rabbits acutely exposed to high altitude, where coagulation is deranged. This is the kind of context-specific in vivo validation needed to translate laboratory materials into field- and clinic-ready haemostats [64]. Together, these studies outline a clear trajectory for zeolite haemostats—from loose granular powders toward firmly anchored, multifunctional and clinically validated devices that retain the intrinsic procoagulant advantages of zeolites while mitigating heat injury, component leaching and infection. From a broader perspective, these studies address, within the field of wound care, the very same fundamental issue that recurs throughout this review: the physical and chemical integrity of zeolite during use. In haemostasis it takes the form of particle detachment from the dressing, with the attendant risk of embolization and distal thrombosis, and of the exothermic hydration that injures tissue; in biomolecule separation and sensing it reappears as framework stability under repeated adsorption–elution and regeneration cycles, and in theranostics as biodegradation and long-term fate in vivo. Anchoring strategies, composite architectures and pre-hydration are, in this light, not merely engineering conveniences but ways of keeping the active material where it is intended to act.

Across these studies, efficacy is most often benchmarked in acute, single-injury animal models over short observation windows, so the durability of the seal, the fate of detached particles and the long-term tissue response remain poorly characterized. Comparability is further limited by heterogeneous end points (haemostasis time, blood loss, survival) and by the frequent absence of standardized positive controls, which makes it difficult to rank formulations against one another.

### 2.2. Separation, Immobilization, and Sensing of Biomolecules

Beyond haemostasis and wound healing, zeolites and zeolite-based materials have attracted growing interest as platforms for the separation, immobilization and detection of biomolecules. This interest stems from their combination of high surface area, tunable surface charge, ion-exchange ability, chemical robustness, and amenability to post-synthetic functionalization, which can be used to control their interaction with proteins, enzymes, nucleic acids, and biological entities dispersed in complex matrices such as blood, plasma, or cell lysates. Crucially, this field cannot be understood in terms of intracrystalline microporosity alone. Many biomacromolecules are larger than the pore apertures of conventional zeolites, so their interaction is governed mainly by external surface area, interparticle mesoporosity, surface charge, Si/Al ratio, hydrophilic/hydrophobic balance, and surface functional groups rather than by diffusion through the framework. For this reason, the most promising biomedical strategies increasingly rely on nanosized zeolites [65], hierarchical or mesoporous architectures [66], magnetic zeolite-derived composites [67], and zeolite-like frameworks integrated with conductive, fluorescent [68], or magnetic components. In this sense, zeolites are best viewed as biointerface materials that can adsorb and stabilize biomolecules, assist their selective recovery, support enzyme immobilization, and serve as inorganic components in biosensing platforms [69].

The diagnosis and monitoring of many diseases increasingly rely on isolating and detecting specific biomolecules from complex biological matrices [70,71]. Proteins, enzymes, nucleic acids, and cell-derived components are key biomarkers in infectious disease, cancer, genetic disorders and inflammation, yet they are often present at low concentration within fluids rich in interfering species—abundant plasma proteins, salts, lipids, metabolites, cells and degradative enzymes. Biological samples are also dynamic, crowded environments in which proteins may undergo conformational change [72], denaturation, or aggregation [73,74]; nucleic acids are prone to enzymatic degradation [75,76]; and low-abundance biomarkers can be masked by albumin, immunoglobulins, or cellular debris [77,78,79]. Efficient separation and stabilization are therefore essential to preserve molecular information before detection [80,81].

**Figure 4:**
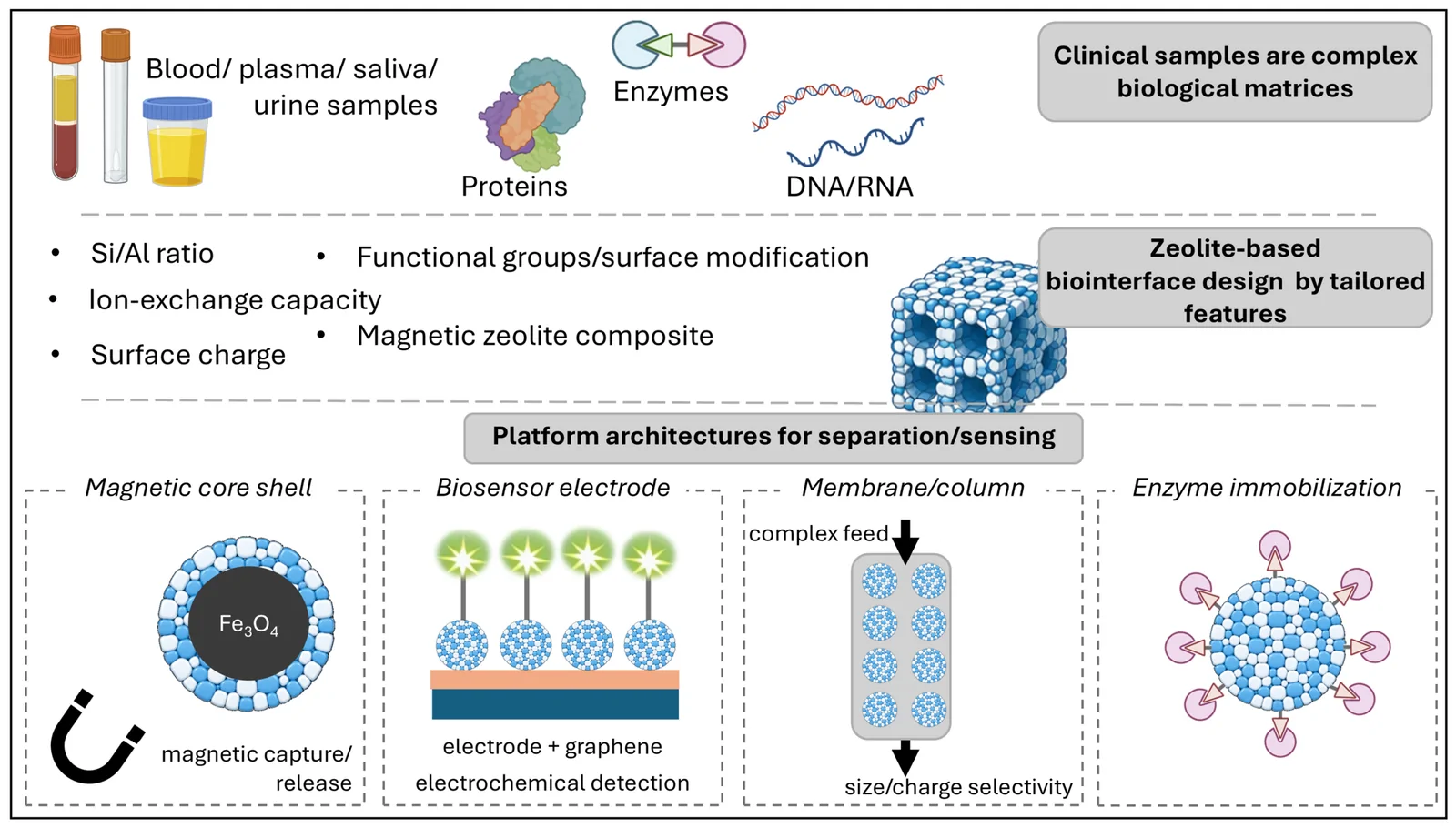
Zeolite-based materials as biointerface platforms for biomolecule separation, immobilization, and sensing. The relevant interactions are mediated by surface charge, ion-exchange sites, hierarchical/interparticle porosity, and surface functional groups; these interactions can be exploited for selective adsorption and preconcentration, enzyme/protein immobilization or refolding, nucleic-acid extraction, and optical/electrochemical biosensing.

Protein purification is a critical first step not only in biomedical and pharmaceutical research but also for downstream technologies such as bioreactors, biosensors, and biofuel cells, which makes the development of effective adsorbents an important goal [32]. Conventional purification and fractionation methods—centrifugation [82], precipitation [83], chromatography [84], dialysis [85], and silica-based extraction [86]—are widely used but often require multiple handling steps, relatively large sample volumes, and careful control of conditions. This increases the risk of sample loss, contamination, or degradation, particularly for small volumes or low-abundance targets. Against this background, zeolites and zeolite-based materials offer a versatile solid-phase platform whose tunable surface charge, ion-exchange capacity, high surface area, and functionalizable external surface can be exploited to adsorb, stabilize, preconcentrate, or selectively release biomolecules (Figure 4).

Protein adsorption on zeolites depends strongly on pH, protein isoelectric point, ionic strength, zeolite composition, and framework hydrophobicity. An early demonstration of these principles was reported by Klint and Eriksson, who studied protein adsorption on ultrastable zeolite Y (USY, Si/Al > 240) and found binding to be greatest at or just below the isoelectric point of the protein [23]. Since the micropores (2.5–7.5 Å) are far too narrow for proteins to enter, adsorption is confined to the external surface, which accounts for only about 1% of the total BET area (roughly 6–7 m^2^/g out of 600–700 m^2^/g); the amount bound nevertheless exceeded this estimated external monolayer about tenfold, leading the authors to propose a first protein layer firmly anchored to the zeolite with further layers retained by protein–protein interactions that can be disrupted by changing pH or ionic strength. On this basis, USY was used in two complementary purification modes—removing unwanted proteins from a crude extract, or adsorbing the target protein and eluting it by lowering the pH—illustrated respectively by peroxidase from horseradish extract and by lysozyme from egg white, the latter with a 16-fold enrichment. Notably, the strongly bound first layer is not released by a pH change. Lysozyme recovery therefore did not exceed about 70% unless the USY was precoated with the same protein, which restored quantitative recovery. The authors also caution that USY and related silicates are unstable at alkaline pH, and recommend avoiding incubation and elution at pH 9 and above. A representative example of selective adsorption and release was reported by Chiku et al., who studied the effect of pH on protein desorption from various zeolites [29]. Testing zeolites of different Si/Al_2_ ratio, they found that bovine serum albumin (BSA) at its isoelectric point (pI) adsorbs most strongly on the zeolites with the highest Si/Al_2_ ratio (H-USY, K-ferrierite/K-FER and zeolite beta/Na-BEA, with Si/Al_2_ ratios of 13.7, 17.7 and 27.4), in agreement with earlier reports [87]. The pI could not be used for desorption with common eluents, so the authors introduced polyethylene glycol (PEG) as an alternative eluent that promotes desorption at the pI without compromising protein activity, exploiting its ability to stabilize protein structure, reduce aggregation, and enhance refolding [88,89]. Using Na-BEA as a model, they showed that different pH values selectively release single proteins from a mixture of ovalbumin (pI 4.6), hemoglobin (pI 7.0), and cytochrome c (pI 10.1): with PEG, ovalbumin eluted at pH 4.3, haemoglobin at pH 7.0, and cytochrome c at pH 10.5. This work is significant because it shows that zeolites can act not only as non-specific adsorbents but also as selective platforms for protein fractionation. Read side by side, these two studies also expose a practical tension that is seldom made explicit: the pH window needed for selective elution may conflict with the chemical stability of the framework itself. Klint and Eriksson note the sensitivity of USY and related silicates to alkaline media and suggest keeping incubation and elution below pH 9, while other schemes operate on different frameworks at higher pH, as in the fractionation of Chiku et al., where cytochrome c is recovered at pH 10.5. The two sets of conditions are not necessarily in conflict, since the materials (highly dealuminated USY versus zeolite beta), contact times and buffer compositions differ; they do indicate, however, that framework stability under the elution conditions should be treated as a design parameter rather than an afterthought, because partial dissolution or dealumination during alkaline steps would compromise reusability, release silicate and aluminate species into the recovered protein fraction, and undermine batch-to-batch reproducibility. Systematically reporting framework integrity after use—for instance by X-ray diffraction, post-use Si/Al analysis or dissolved-silicon measurements—would place the comparison of such strategies on a firmer basis.

**Figure 5:**
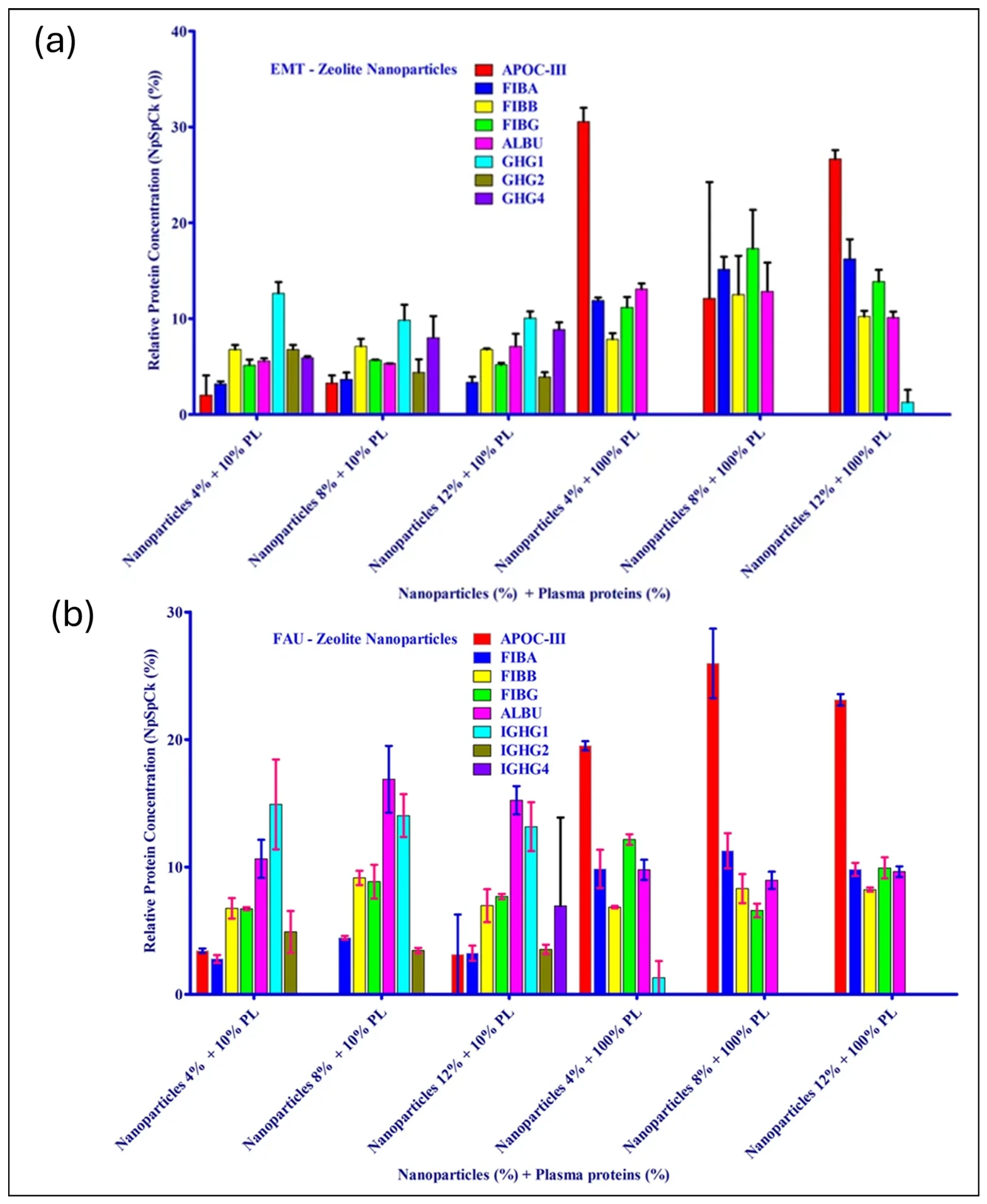
Selective sorption of human plasma proteins on nanosized zeolites. Relative concentration of the principal adsorbed plasma proteins on (**a**) EMT-type and (**b**) FAU-type zeolite nanoparticles, as a function of nanoparticle content (4, 8 and 12%) and plasma dilution (10% and 100%). Albumin and immunoglobulin G dominate the corona in diluted plasma, whereas apolipoprotein C-III and fibrinogen chains become prominent at higher plasma concentrations, illustrating how framework type, external surface chemistry and the zeolite-to-plasma ratio jointly determine the composition of the adsorbed protein layer. Reproduced from Rahimi et al. [27].

Rahimi and co-workers highlighted the relevance of zeolite–protein interactions in biological fluids, studying the selective sorption of nanosized EMT- and FAU-type zeolites for human plasma proteins (Figure 5) [27]. They identified three key parameters: plasma concentration, the type of zeolite (external surface area and hydrophilicity), and the amount of zeolite nanoparticles. Albumin and immunoglobulin G dominated the protein corona in diluted plasma, whereas apolipoprotein C-III and fibrinogen chains became relevant at higher plasma concentrations—relevant because protein adsorption in realistic fluids governs colloidal stability, biological identity, immune recognition, and downstream separation efficiency. These data are worth examining in detail (Figure 5), because they provide one of the few quantitative demonstrations that the composition of the adsorbed protein layer—and therefore the biological identity the particle acquires—can be steered by framework type and by the zeolite-to-plasma ratio alone, without any surface functionalization: the shift in corona composition described above. This is precisely the kind of systematic, condition-resolved reporting advocated in Section Toward Standardized Reporting of Bio-Interface Interactions, and it illustrates why comparisons drawn from single-protein experiments can be misleading. The dominant role of zeolite hydrophilicity was also shown by Mesgari-Shadi et al., who used zeolites to purify dilute scFv solutions expressed in *E. coli* [90]. Matsui et al. examined potassium-form zeolites (K-LTL and K-FER) for protein adsorption and desorption [32]: K-LTL, with a low SiO_2_/Al_2_O_3_ ratio and large pores, adsorbs and releases basic proteins such as chymotrypsinogen A and lysozyme well, whereas K-FER, with a high SiO_2_/Al_2_O_3_ ratio and small pores, binds all proteins firmly. The practical weight of these adsorption phenomena is not confined to the laboratory: natural zeolites have been evaluated as an alternative to bentonite for removing haze-forming proteins during white-wine stabilization, an industrial-scale separation in which adsorption selectivity and product losses carry direct economic consequences [91].

Zeolites are also of interest as protein-adsorbing supports for the recovery of recombinant proteins. Overexpression in host systems often yields inactive, insoluble aggregates (inclusion bodies) that must be denatured and then refolded by removing the denaturant [92]. Dilution is the most common refolding route because of its simplicity, but it requires large buffer volumes and low protein concentrations to prevent aggregation. Adsorbing proteins onto a solid support is an attractive alternative that reduces buffer volume and limits aggregation [93].

**Figure 6:**
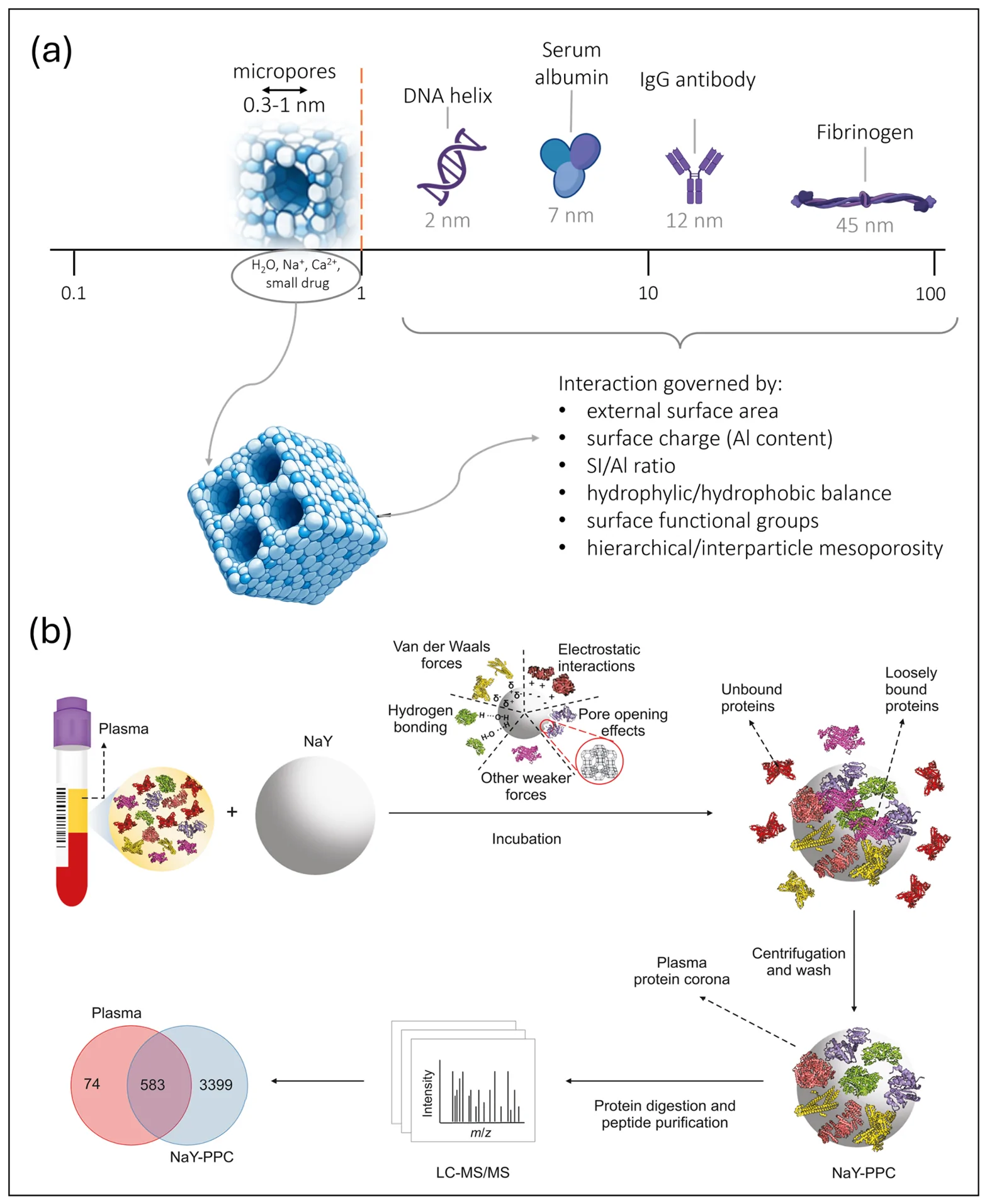
Molecular mechanisms of zeolite–protein interaction in biological fluids. (**a**) Zeolite pore aperture versus biomolecule size: micropores admit only small species, whereas proteins and nucleic acids interact at the external surface rather than by intracrystalline sieving; the enlarged view summarizes the forces that operate there (electrostatic, hydrogen-bonding, van der Waals, hydrophobic, and pore-opening effects). (**b**) Protein-corona formation on the zeolite NaY surface and the associated proteomic workflow, exploited to enrich low-abundance plasma proteins for deep proteome profiling; panel (**b**) is reproduced from Ma et al. [24].

Overall, protein separation on zeolites cannot be rationalized by molecular sieving alone; it reflects a combination of electrostatic interactions, hydrogen bonding, hydrophobic effects, and competitive adsorption among plasma proteins (Figure 6a). This is central to the design of zeolite-based bioseparation systems, because protein-corona formation can be either a limitation—causing fouling and loss of selectivity—or an opportunity, when deliberately exploited for selective enrichment of target proteins [94]. A recent study illustrates this opportunity directly: the plasma protein corona formed on zeolite NaY was used as an enrichment strategy for proteomic analysis, enhancing the detection of low-abundance plasma proteins by reducing the masking effect of highly abundant species and allowing about 4000 proteins to be identified in a single LC-MS/MS run (Figure 6b) over a dynamic range of roughly eight orders of magnitude [24].

The immobilization of proteins and enzymes on solid supports improves reusability, operational stability, and ease of separation from products [95,96,97,98,99]. Zeolites are attractive supports because they combine chemical and thermal stability with tunable acidity/basicity, ion-exchange capacity, and high surface area; immobilization can occur through physical adsorption, electrostatic interactions, covalent anchoring, encapsulation in secondary porosity, or attachment to surface functional groups. The small micropores of classical zeolites, however, limit the accessibility of large enzymes, which is why hierarchical, delaminated, hollow, or nanozeolitic architectures are increasingly preferred for biocatalysis [66]; a recent review emphasizes that mesoporosity and external-surface engineering are key to improving mass transfer and preserving enzyme activity [100].

Becker and colleagues developed a mild nitroxide-exchange reaction to site-selectively immobilize dyes and proteins within zeolite L crystals [33]. The crystals were functionalized with carboxylic-acid-bearing alkoxyamines that form amide bonds with amine groups placed either at the channel entrances or across the whole surface. Using rhodamine and nitrobenzoxadiazole as fluorescent probes, and then biomolecules such as mannose, biotin, and L-lysine, they achieved efficient, position-selective binding, with opportunities in targeted delivery and sensing.

Zeolites have also been used as protein-adsorbing supports for refolding. Nara and co-workers used zeolite beta to refold lysozyme, a disulfide-bonded protein [30]: denatured hen-egg lysozyme was adsorbed onto Na-exchanged zeolite beta, with adsorption plateauing after ~60 min. Adding a small volume of refolding buffer (redox reagents, guanidine hydrochloride, PEG, and L-arginine) then desorbed the protein rapidly (plateau at ~2 h), while activity recovered more slowly and peaked after ~16 h, yielding active lysozyme at high concentration. The concept was later extended to the refolding and reassembly of PEGylated protein nanoparticles using zeolite beta and zeolite L without conventional denaturants, with potential applications in drug delivery, vaccine development, and nanomaterials synthesis [101]. Together, these examples suggest that zeolites can serve as temporary inorganic reservoirs that modulate local protein concentration and reduce aggregation, although optimization remains highly protein-specific.

A second major direction is nucleic acid extraction and purification, a mandatory step in molecular diagnostics, forensic analysis, genetic engineering, and pathogen detection [102]. Conventional liquid-phase protocols rely on repeated precipitation, centrifugation, and washing, which are time-consuming and labour-intensive and increase the risk of degradation, loss, or cross-contamination. Solid-phase strategies address several of these drawbacks by capturing nucleic acids on a recoverable surface through hydrogen bonding, electrostatic interactions, ion exchange, affinity, and size-exclusion effects [103]; because easy recovery of the solid is essential, magnetic solid adsorbents are especially attractive.

Pansini and co-workers prepared magnetic metal–ceramic nanocomposites by thermally treating Fe-exchanged zeolites A or X under a reducing atmosphere, and used them as magnetic adsorbents to separate the target genetic factors V and RNASE and *Staphylococcus aureus* DNA from human blood [22]. Compared with a commercial magnetic separation system, the zeolite-derived adsorbents performed similarly or better with an appropriate protocol, particularly for small blood volumes—an important step from model adsorption experiments toward biologically complex matrices. The concept was recently extended to RNA extraction from the blood of pediatric patients with respiratory syncytial virus (RSV) bronchiolitis, where zeolite-derived magnetic nanocomposites were compared with a commercial silica-column system, suggesting a low-cost, scalable alternative for molecular-diagnostic workflows [104]. Although validation on larger cohorts and standardized protocols is still needed, these studies indicate that zeolite-derived magnetic adsorbents could be useful in diagnostic sample preparation when small volumes, magnetic handling, and reduced complexity are desirable.

It is worth noting that, in this area, zeolites often act not as the final crystalline microporous adsorbent but as precursors for porous magnetic metal-ceramic composites; the biomedical function then derives from the combination of zeolite-derived porosity, surface chemistry, and magnetic phases rather than from the framework alone.

The same properties that support bioseparation and immobilization make zeolites attractive in biosensors: their high surface area increases probe or enzyme loading, their ion-exchange ability can preconcentrate charged analytes, their porous structure provides a confined microenvironment, and their surface can be coupled with conductive materials such as graphene, carbon nanotubes, or metal nanoparticles to improve signal transduction [105]. A 2022 review surveyed zeolites and zeolitic imidazolate frameworks (ZIFs) in electrochemical and optical biosensing based on enzymes, antibodies, DNA, and aptamers [69]. ZIFs are metal–organic frameworks rather than aluminosilicate zeolites, but their zeolite-like topology and extensive use in biosensing make them useful comparators.

An example based on conventional zeolites was reported by Narang and co-workers, who built an impedimetric genosensor for ultratrace detection of hepatitis B virus DNA in blood using a nanocomposite of zeolite nanocrystals and multi-walled carbon nanotubes [106]. The zeolite provided chemical stability and a large surface area for DNA immobilization, while the carbon nanotubes improved conductivity and electron transfer; the device reached ~99% detection accuracy and could be regenerated by brief immersion in a hydroxide solution. This illustrates a recurring principle: zeolites are frequently combined with conductive or electroactive phases to overcome their intrinsically insulating nature.

Recent zeolite-like platforms further illustrate this diagnostic potential. A ZIF-8-based fluorescent biosensor was reported for rapid COVID-19 RNA detection [107]: ZIF-8 quenched the fluorescence of a FAM-labelled probe DNA through electrostatic and π–π interactions, and hybridization with the complementary RNA restored the signal, giving a limit of detection of 6.24 pM, an 8 min assay, and selectivity against mismatched sequences. Electrochemical sensing has also benefited from zeolite composites: a Cu–Fe nanoparticle/zeolite A/graphene electrode detected dopamine with a limit of detection of 0.058 μM and a sensitivity of 1.97 μA μM^−1^ cm^−2^ [108], with zeolite A providing structural support and ion-exchange/adsorption sites, graphene improving conductivity, and the bimetallic nanoparticles enhancing electrocatalysis. It should be noted, however, that these analytical performance parameters are typically obtained under optimized conditions—single analytes in clean buffers—and that detection limits and sensitivities reported by different groups are difficult to compare directly, since they depend on electrode preparation, the zeolite loading, and the measurement protocol; validation in real biological matrices, where competitive adsorption and fouling intervene, is reported far less often than the idealized performance.

#### Toward Standardized Reporting of Bio-Interface Interactions

Overall, the use of zeolites in biomolecule separation, immobilization and sensing highlights their role as biointerface materials that control molecular recognition, stabilization and signal generation—the same functions that underpin the more advanced theranostic platforms discussed next.

Much of the evidence in this area rests on single-protein model systems, whereas performance in real biological fluids is governed by competitive, time-dependent adsorption—the Vroman effect—in which initially bound abundant proteins are progressively displaced by higher-affinity species. This dynamic exchange directly affects the reproducibility of targeted biomarker isolation from plasma and is rarely quantified. Reported outcomes also depend on variables that are inconsistently documented across studies—Si/Al ratio, ionic strength, surface-charge distribution, protein conformational state on adsorption, and desorption yield—so that apparently conflicting reports of reversible versus irreversible binding often reflect differences in testing conditions rather than in the materials themselves. Systematic reporting of these parameters, ideally against a common set of test matrices, would place the comparison of bio-interface interactions on a firmer footing.

The reproducibility problem deserves particular emphasis for biomarker isolation, one of the most promising uses of zeolites in this area. In plasma, the adsorbed layer is not fixed but evolves: the Vroman effect describes how abundant, fast-diffusing proteins such as albumin adsorb first and are then progressively displaced by less abundant, higher-affinity species, so that the composition of the captured layer depends on incubation time, plasma dilution, and the surface chemistry of the sorbent [109,110]. For targeted capture, this is a double-edged property: the same competitive displacement that can enrich a low-abundance marker by shedding albumin can also, under slightly different conditions, strip the target itself. Because the corona forms within seconds and reorganizes over minutes to hours, small, undocumented differences in protocol translate into large differences in which proteins are recovered, which is a principal reason why reports of zeolite-based biomarker enrichment are difficult to reconcile [111].

We therefore suggest that studies of zeolite bio-interface interactions converge on a common reporting protocol, so that results can be compared rather than merely collected. At a minimum, we recommend that each study specify:

- The material descriptors that govern adsorption: framework type, Si/Al ratio, particle size and external surface area, and surface charge (zeta potential) under the test conditions;
- The solution conditions: pH, ionic strength and buffer composition, protein or analyte concentration, temperature, and incubation time;
- The matrix: whether a single-protein solution, a defined multi-protein mixture, or a real fluid (serum, plasma) is used, with the dilution stated;
- The outcome metrics: adsorption capacity, desorption/elution yield, and, where possible, evidence on the conformational state and retained activity of the released biomolecule.

Reporting these variables against a small set of shared reference matrices would resolve much of the present ambiguity between reversible and irreversible binding, which frequently reflects differences in testing conditions rather than in the materials themselves, and would make the comparison of bio-interface interactions quantitative rather than anecdotal.

### 2.3. Theranostic Applications of Zeolite-Based Materials

The growing demand for personalized medicine has driven the development of multifunctional platforms that combine diagnosis, therapy, and treatment monitoring in a single material. This concept—*theranostics*—integrates therapeutic and diagnostic functions in the same agent [112,113,114,115]. In an ideal system, the diagnostic component enables disease localization, target identification, or real-time monitoring of biodistribution, while the therapeutic component provides drug delivery, ion release, photodynamic/photothermal action, magnetic hyperthermia, or other disease-modulating effects. This is especially attractive in oncology [116], infectious disease [117], inflammatory disorders [118], and degenerative pathologies [119], where early detection, local treatment, and monitoring improve outcomes.

Zeolites and zeolite-based materials are promising, though still relatively underexplored, candidates for theranostic design, for the same reasons discussed above: high surface area, tunable porosity, ion-exchange capacity, chemical stability, external-surface reactivity, and the possibility of hosting or anchoring functional species. These properties let zeolites host therapeutic molecules, exchange bioactive ions, immobilize imaging probes, and integrate with magnetic, fluorescent, or polymeric components [120]. For example, fluorescent dye-loaded faujasite-type zeolites have been explored as bioimaging tools, showing cell compatibility and traceability by confocal microscopy [25], and recent reviews highlight zeolite-based nanoparticles as drug-delivery systems with structural tunability, biocompatibility, and controlled release for antitumour, antibacterial, and imaging applications [121,122].

**Figure 7:**
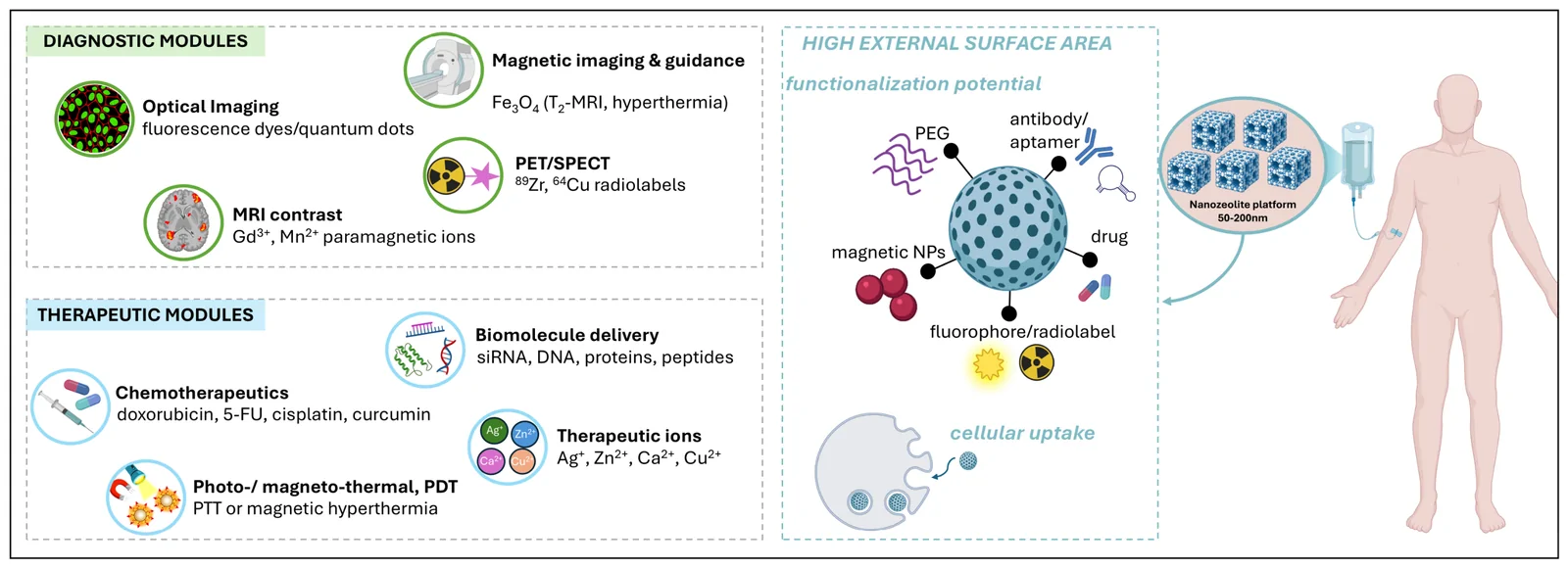
Nanosized zeolites as multifunctional theranostic platforms. Diagnostic modules (green) include optical imaging (fluorescence dyes and quantum dots), MRI contrast (Gd^3+^, Mn^2+^ paramagnetic ions), magnetic imaging and guidance (Fe_3_O_4_, enabling T_2_-MRI and hyperthermia) and PET/SPECT radiolabels (^89^Zr, ^64^Cu); therapeutic modules (blue) include chemotherapeutics (doxorubicin, 5-FU, cisplatin, curcumin), biomolecule delivery (siRNA, DNA, proteins, peptides), therapeutic-ion release (Ag^+^, Zn^2+^, Ca^2+^, Cu^2+^) and photo-/magneto-thermal therapy and PDT. The magnified inset highlights the three features that distinguish nanosized from bulk zeolites: a high external surface area, surface functionalization with PEG, targeting ligands (antibodies, aptamers), drugs, fluorophores/radiolabels, and magnetic nanoparticles, and efficient cellular uptake across biological barriers. Created in BioRender. Tammaro, O. (2026) https://BioRender.com/5a6nth0 (accessed on 4 September 2026).

A crucial requirement is the transition from bulk or micrometric zeolites to nanozeolites [123,124]. Conventional zeolites are widely used as adsorbents, ion exchangers, and haemostatic agents, but their large particle size limits systemic use where cellular uptake, tissue penetration, colloidal stability, and controlled biodistribution are required [125]. Reducing crystals to the nanoscale increases the external surface-to-volume ratio and exposes more accessible surface sites [65]—particularly relevant because many therapeutic molecules, proteins and nucleic acids are too large to enter the micropores (Figure 7).

From a theranostic standpoint, nanozeolites offer several advantages. Their high external surface area enables efficient functionalization with targeting ligands, polymers, fluorescent probes, radiolabels, magnetic nanoparticles, or biomolecules—essential for integrating diagnostic and therapeutic functions in one platform. Their nanoscale size can favour interaction with cells and biological barriers, allowing intracellular delivery or cell-associated imaging depending on size, surface charge, shape, and coating [125,126]. Studies on surface-functionalized nanozeolites show that uptake and cytotoxicity depend strongly on surface properties as well as composition, underscoring the need for rational surface engineering [127]. Nanosized zeolites also disperse more readily in aqueous media, although colloidal stabilization with polymers, surfactants, or biomimetic coatings is often required to prevent aggregation in physiological fluids [128].

The crystalline framework can also act as a robust inorganic host for diagnostic and therapeutic components: paramagnetic ions, radionuclides, fluorescent dyes, or photosensitizers can be incorporated or anchored, while therapeutic molecules can be loaded on the accessible surface, within hierarchical porosity or inside suitable channels. For instance, nanosized Linde type L zeolites have been explored as multimodal imaging platforms by incorporating gadolinium for MRI and radiolabelling with zirconium-89 for PET [34].

A particularly relevant strategy is the development of magnetic zeolite nanocomposites, which combine the loading capacity and surface chemistry of zeolites with the magnetic properties of iron oxide nanoparticles, potentially integrating drug delivery, magnetic recovery, MRI, and magnetic hyperthermia. A NaY-based magnetic nanocomposite containing magnetite was validated in vitro as a T_2_-MRI contrast agent, showing superparamagnetic behaviour, internalization by breast cancer cells, low cytotoxicity under the tested conditions, and enhanced dark contrast at a clinical field of 3 T [129]—directly linking the magnetic zeolite-derived materials of the previous section to image-guided therapeutic design.

In cancer therapy, zeolite-based systems have been investigated as carriers for anticancer agents such as doxorubicin, 5-fluorouracil, curcumin, cisplatin, and nucleic-acid therapeutics. A systematic review evaluated zeolites and ZIFs as drug-delivery systems for anticancer treatment, highlighting improvements in drug loading, release control, and performance [26]. ZIF-8 nanoplatforms in particular have been widely studied for cancer delivery, pH-responsive release and image-guided therapy, though their clinical translation still requires careful evaluation of stability, toxicity, biodegradation, and long-term fate [130].

Therapeutically, zeolite-based systems can support several strategies. In conventional chemotherapy, they mainly improve drug solubility, protect the cargo, reduce premature degradation, and promote localized release. In photodynamic therapy, they can carry photosensitizers, improving dispersion and limiting aggregation. In photothermal or magnetothermal approaches, composites with optically or magnetically active components can contribute to local heat generation; magnetic clinoptilolite-type nano/microparticles, for example, have been explored as multifunctional systems for controlled release, imaging and local heating using photosensitizer-like molecules such as sulfonated aluminum phthalocyanine and hypericin [35]. A caveat must frame this entire section: many of the most advanced systems below are ZIFs, that is, metal–organic frameworks with zeolite-like topologies rather than aluminosilicate zeolites, and the two classes must not be conflated (Section 2.3.1 and Table 2). Representative systems of this kind, together with the diagnostic and therapeutic functions they combine, the models in which they were validated, and the main outcomes reported, are summarized in Table 3 and Table 4.

Despite these examples, translation remains challenging. The very features that make zeolites attractive—high surface area, surface charge, ion exchange, and strong adsorption—also complicate their biological behaviour, so particle size, morphology, colloidal stability, protein-corona formation, biodistribution, biodegradability, clearance, dose-dependent toxicity, and long-term accumulation must be carefully controlled. The microporous nature of conventional zeolites further limits the loading of large therapeutics, making hierarchical porosity, external-surface functionalization, and composite design essential; recent reviews explicitly stress the need for safety assessment and a deeper understanding of nanoparticle–biological interactions before clinical use [122]. A further limitation is apparent from Table 3 and Table 4 themselves: with the notable exceptions of the upconversion nanocomposite of Zheng et al. [131] and the ZSM-5/chitosan nanodisks of Yang et al. [28], both of which report efficacy in tumour-bearing animals, the systems that have reached in vivo validation are almost exclusively ZIF, and among true aluminosilicate zeolites most studies stop at cell-culture experiments or at biodistribution in healthy animals. Reports that combine a genuine diagnostic readout with a demonstrated therapeutic effect in a disease model, and that follow the material beyond the acute phase, remain scarce. This imbalance is informative in itself: the theranostic potential of aluminosilicate zeolites is still largely projected from their physicochemical properties rather than demonstrated in vivo. Closing this gap is arguably the most pressing requirement for the field, and it will require studies in tumour-bearing or infection models that report efficacy, quantitative biodistribution, clearance, and comparison with the free agent.

**Table 2:** Structural and physicochemical comparison of aluminosilicate zeolites and zeolitic imidazolate frameworks (ZIFs). The two classes share only a topological analogy; their composition, bonding, degradation pathways and in vivo behaviour differ fundamentally, which is why their translational status is not interchangeable. Compiled from [3,13,132,133,134]: framework composition and topology from Park et al. and the IZA-SC database; framework flexibility and gate-opening behaviour from Fairen-Jimenez et al.; degradation behaviour in physiological media from Velásquez-Hernández et al. and Abdelhamid.

Property	Aluminosilicate Zeolite	Zeolitic Imidazolate Framework (ZIF)
**Material class**	Purely inorganic tectosilicate	Metal–organic framework (MOF) subclass
**Building units**	SiO_4_ and AlO_4_ tetrahedra	Divalent metal ions (e.g., Zn^2+^) + imidazolate linkers
**Bonding**	Covalent Si–O–Al network	Metal–nitrogen coordination bonds
**Framework rigidity**	Rigid, thermally very stable	More flexible; “gate-opening” and linker dynamics
**Behaviour in vivo**	Essentially non-biodegradable at physiological pH	Acid-labile; disassembles in the tumour/endo-lysosomal environment
**Degradation trigger**	None built in; slow, non-specific dissolution (mainly alkaline)	Protonation of imidazolate linkers → framework collapse
**Degradation products**	Soluble silicic and aluminic species	Metal ions (e.g., Zn^2+^) + 2-methylimidazole
**Stimuli responsiveness**	Limited; not intrinsic	Intrinsic pH-responsiveness exploited for triggered release
**Clearance route**	Slow surface erosion; reticuloendothelial sequestration	Dissolution into metabolizable/excretable species
**In vivo theranostic evidence**	Sparse; largely in vitro or healthy-animal	Extensive; tumour-bearing-animal validation common

#### 2.3.1. Aluminosilicate Zeolites Versus ZIFs: A Necessary Distinction

Classical zeolites are purely inorganic aluminosilicates: a rigid, covalently bonded Si–O–Al framework that is thermally robust and, under physiological conditions, essentially non-biodegradable [3,13]. Zeolitic imidazolate frameworks are not zeolites but a subclass of metal–organic frameworks, built from divalent metal ions (typically Zn^2+^) bridged by organic imidazolate linkers into zeolite-like topologies [3]. The shared topology is purely geometric; the chemistry, and with it the behaviour in a biological environment, is fundamentally different (Table 2) [132].

The organic–inorganic hybrid nature of ZIFs confers a distinct degradation profile. Beyond composition, the hybrid nature of ZIFs also changes the mechanical behaviour of the framework. Whereas an aluminosilicate lattice is rigid and its aperture essentially fixed, the metal–imidazolate coordination bonds are comparatively labile, and the organic linkers can rotate or tilt, giving rise to “gate-opening” and other framework-flexibility phenomena that modulate guest uptake and release in ways that a covalent Si–O–Al network cannot [134]. Under the mildly acidic conditions of the tumour microenvironment or the endo-lysosomal compartment, protonation of the imidazolate linkers weakens the metal–nitrogen coordination bonds, the framework disassembles, and the constituents—Zn^2+^ ions and 2-methylimidazole—are released and can enter normal metabolic or excretory pathways. This acid-triggered collapse is precisely what makes ZIFs attractive as stimuli-responsive carriers, since it couples cargo release to the target environment [133,135]. Aluminosilicate zeolites possess no comparable built-in degradation switch: their framework dissolves only slowly and non-specifically, chiefly under strongly alkaline conditions, and any structural change is more a stability liability during elution or regeneration than a designed therapeutic trigger.

This distinction has direct consequences for translation, and it should be read as a caveat against transferring ZIF results onto aluminosilicates. The advanced in vivo theranostic literature is dominated by ZIFs, whose stimuli-responsive degradation and clearance are comparatively well characterized, whereas aluminosilicate zeolites remain largely bounded by in vitro or healthy-animal evaluations. The successes of ZIF platforms should therefore be read as structural and conceptual inspiration for zeolite design—not as direct evidence that aluminosilicate zeolites will behave similarly in vivo, given their non-biodegradability and different clearance behaviour.

#### 2.3.2. Clearance, Accumulation, and Long-Term Safety of Nanozeolites

A specific aspect of aluminosilicate nanozeolites, and largely absent from the primary literature surveyed above, is their long-term fate once introduced systemically. Being rigid, non-biodegradable inorganic particles, nanozeolites are expected to follow the clearance behaviour documented for related silica and mesoporous-silica nanoparticles: rapid removal from the circulation by the reticuloendothelial system, with passive sequestration and prolonged retention in the liver and spleen, and a clearance rate governed by particle size, aspect ratio, surface charge, and porosity rather than by chemical dissolution [136]. As the structure does not readily dissolve at physiological pH, elimination depends on slow surface erosion to soluble silicic and aluminic species followed by renal excretion, so that a substantial fraction of the administered dose may remain sequestered in reticuloendothelial organs for weeks to months.

This slow, essentially passive clearance carries safety implications that scale with dose. Chronic-toxicity studies of intravenously administered silica nanoparticles have reported immunotoxicity manifesting months after the particles themselves were cleared, underscoring that the absence of acute toxicity does not guarantee long-term safety [137]. The mechanism linking particle size to clearance runs through the protein corona. Because biological responses to nanoparticles scale with surface area rather than mass, increasing the surface-to-volume ratio—the very step that makes nanozeolites attractive for functionalization—amplifies the adsorption of plasma proteins, and in particular of opsonins such as immunoglobulins, complement fragments, and fibrinogen, whose “molecular signature” marks the particle for recognition and engulfment by mononuclear phagocytes [111,138]. Denser opsonization accelerates macrophage uptake and hepatic and splenic sequestration, shortening circulation time and concentrating the dose in reticuloendothelial organs; surface chemistry, charge, and any adsorbed dysopsonins (e.g., albumin) modulate but do not remove this effect. This route is saturable: the mononuclear phagocyte system has a finite uptake capacity, so that above a threshold dose clearance kinetics change and particles persist longer in the circulation and in already-loaded organs, compounding the retention problem [139]. Aggregation in physiological fluids raises a further risk of capillary embolization. Taken together, dose-dependent accumulation limits, reticuloendothelial saturation thresholds and the potential for chronic inflammation should be treated as first-order design constraints for any systemic nanozeolite, and evaluated with the same rigour as therapeutic efficacy [136].

A second, cell-level dimension of the same problem is intracellular persistence. Nanoparticles taken up by macrophages and other cells are trafficked to the endo-lysosomal compartment, where an organic or metal–organic carrier would normally be degraded; a rigid aluminosilicate framework, by contrast, is only slowly attacked even at lysosomal pH and can therefore accumulate within cells over repeated exposures. Persistent, poorly degradable inorganic particles are a recognized trigger of “frustrated” phagocytosis, lysosomal destabilization, and sustained release of pro-inflammatory mediators [140], and this is precisely where particle morphology and biopersistence—rather than bulk composition—become decisive. High-aspect-ratio or non-clearable nanozeolites therefore warrant particular scrutiny, and their intracellular fate should be documented alongside organ-level biodistribution.

It is useful to place this behaviour on a common scale of clearability. At one extreme, polymeric and lipidic carriers are engineered for programmed breakdown—hydrolysis of ester or amide bonds, or enzymatic cleavage—into small, excretable fragments on a time scale matched to their therapeutic task. ZIFs and related metal–organic frameworks occupy an intermediate position: they are not spontaneously metabolized, but their coordination bonds hydrolyse, and their linkers protonate under mildly acidic conditions, so that they disassemble into metabolizable ions and small organic molecules in the very compartments where they act. Aluminosilicate zeolites sit at the opposite extreme: the Si–O–Al network offers no built-in hydrolytic or pH-triggered cleavage pathway, so clearance relies almost entirely on slow, non-specific surface dissolution. This gradient—from designed degradability, through stimulus-triggered disassembly, to near-inertness—explains why the safety questions that are secondary for degradable carriers become first-order for aluminosilicate nanozeolites, and why clearance behaviour should be regarded as a core design specification rather than an afterthought.

#### 2.3.3. A Methodological Standard: Reporting Framework Stability Under Use

The recurring observation that framework stability is rarely documented (Section 1, Section 2.2 and Section 2.3.1) should, in our view, be turned from a caveat into an explicit reporting standard for the field. Because partial dissolution or dealumination changes surface charge, releases silicate and aluminate species, and can generate cytotoxic leached ions, a material characterized only before use is characterized only halfway. We therefore propose that any study placing a zeolite in contact with biological fluids, alkaline elution buffers, or repeated regeneration cycles report, as a minimum, the following post-use measurements alongside the pre-use characterization:

- Framework integrity and crystallinity by powder X-ray diffraction before and after use, to detect amorphization or phase change;
- The framework Si/Al ratio (e.g., by ICP-OES or EDX) before and after use, to quantify dealumination;
- The concentration of dissolved silicon and aluminum species released into the contacting medium, as a direct measure of framework erosion;
- Where systemic use is intended, the identity and cytotoxicity of the leached species at the concentrations actually reached.

Reporting these quantities would make results comparable across laboratories, expose silent degradation that currently passes unnoticed, and connect material performance directly to the safety concerns discussed in Section 2.3.2. We regard this as a low-cost, high-value requirement that reviewers and editors are well placed to encourage.

The contrast between Table 3 and Table 4 is itself a central finding of this review. Of the seven aluminosilicate-zeolite platforms in Table 3, only two [28,131] report therapeutic efficacy in tumour-bearing animals, whilst the other are limited to in vitro or healthy-animal studies; by contrast, ten of the twelve ZIF platforms in Table 4 were validated in tumour-bearing or infected-animal models. This roughly fivefold disparity in the depth of in vivo validation, concentrated almost entirely in the metal–organic ZIF class rather than in true aluminosilicates, quantifies the translational gap discussed above. In practical terms, this is the principal bottleneck to clinical implementation of aluminosilicate nanozeolites: the clinical case is at present largely inferred from a chemically distinct class, and closing it will require aluminosilicate-specific efficacy, biodistribution, and clearance data in disease models rather than in healthy animals.

**Table 3:** Representative aluminosilicate-zeolite platforms for theranostic applications, summarizing the diagnostic and therapeutic functions combined in each system, the model in which it was validated and the main reported outcome. For therapy-focused entries, the diagnostic column lists the imaging-capable component of the platform even where imaging was not the focus of the study.

Zeolite Platform	Diagnostic Function	Therapeutic Function	Model Tested	Key Reported Outcome	Ref.
PEGylated Gd(III)/^89^Zr-loaded nanozeolite Linde type L (LTL)	MRI (Gd^III^) and PET/CT (^89^Zr)	None demonstrated (imaging and biodistribution only)	Healthy mice, in vivo (i.v. injection)	Initial lung uptake followed by migration to liver and spleen; ex vivo biodistribution confirmed in vivo stability, the original metal/Si ratio being preserved	[34]
NaY–magnetite magnetic nanocomposite	T_2_-weighted MRI	Magnetic guidance and recovery; drug-carrier platform	Breast cancer cells, in vitro	Superparamagnetic behaviour, cell internalization, low cytotoxicity and enhanced dark contrast at a clinical field of 3 T	[129]
Magnetic clinoptilolite nano/microparticles loaded with sulfonated Al-phthalocyanine or hypericin	Fluorescence imaging	Photodynamic action and local (magneto)thermal heating; controlled release	In vitro; ex vivo chicken tissue (magnetic heating)	Controlled release, imaging and local magnetic heating; the particles also showed an anti-amyloidogenic effect on insulin and lysozyme	[35]
Fluorescent dye-loaded faujasite-type zeolite	Confocal fluorescence bioimaging	Host/carrier for functional molecules	Cells, in vitro	Cell compatibility and traceability by confocal microscopy	[25]
Upconversion zeolite-based nanocomposite (lanthanide-exchanged)	NIR-excited upconversion luminescence	Ultrasound- and NIR-dual-triggered hyperthermia with ROS generation (PDT/SDT)	Melanoma, in vitro and in vivo	Hyperthermia-enhanced multimodal therapy acting through a defined apoptotic mechanism	[131]
Mesoporous ZSM-5 zeolite/chitosan core–shell nanodisks loaded with doxorubicin	Not integrated (delivery-focused study)	pH-responsive doxorubicin release (chemotherapy)	MG-63 osteosarcoma cells and tumour-bearing mice	Drug-loading efficiency of 97.7% and markedly enhanced release in acidic medium (58.7% at pH 6); efficient tumour inhibition with reduced cardiac toxicity, confirmed by pharmacokinetic, serological and histological analysis	[28]
Gd^3+^-loaded faujasite NaX zeolite	T_1_-weighted MRI	Imaging agent (carrier-capable framework)	Healthy rats, in vivo (7 T)	Very high longitudinal relaxivity (~52 mM^−1^ s^−1^, well above Gd-DOTA); MR angiography validated in vivo	[141]

**Table 4:** Representative zeolite-like metal–organic frameworks (zeolitic imidazolate frameworks, ZIFs) used in theranostic applications. ZIFs share zeolite topologies but are metal–organic frameworks, not aluminosilicates (see Section 2.3.1); they are tabulated separately to make the contrast with Table 3 explicit.

Zeolite Platform	Diagnostic Function	Therapeutic Function	Model Tested	Key Reported Outcome	Ref.
ZIF-8 loaded with indocyanine green and paclitaxel	NIR fluorescence (indocyanine green)	pH/NIR-responsive release; combined chemo- and photothermal therapy	A549 NSCLC cells; tumour-bearing mice, in vivo	NIR-guided chemo-phototherapy from a single decomposable carrier	[142]
Dextran-coated Cy5.5 and doxorubicin co-loaded ZIF-8	NIR fluorescence imaging (Cy5.5)	Acid-responsive chemotherapy (doxorubicin)	A549 tumour-bearing mice, in vivo	Tumour fluorescence about 48-fold higher than free Cy5.5, with improved efficacy relative to free doxorubicin	[143]
Folate-targeted FZIF-8/DOX with Gd-doped Si nanoparticles and chlorin e6	MRI (Gd) and fluorescence (Ce6) dual-modal	pH-responsive doxorubicin chemotherapy + chlorin e6 photodynamic therapy	Folate-receptor-positive cancer cells, in vitro	Dual-modal imaging-guided chemo/PDT from a single targeted, low-leakage carrier	[144]
Fe_3_O_4_ZIF-8 loaded with doxorubicin	Fe_3_O_4_ (magnetic; MRI/guidance-capable)	pH-responsive doxorubicin chemotherapy	Osteosarcoma cells and tumour-bearing mice, in vivo	Enhanced antitumour effect with reduced drug toxicity, in vitro and in vivo	[145]
DOX-loaded MnO_2_ZIF-8	MnO_2_ (H_2_O_2_/TME-responsive; Mn^2+^ MRI-capable)	Chemotherapy + photothermal therapy	Lewis lung carcinoma, in vivo	~156 nm particles, 12 wt% DOX loading; increased apoptosis and tumour suppression	[146]
Biomimetic DOX/CuSZIF-8 (red-blood-cell membrane + catalase)	CuS (photothermal; photoacoustic-capable)	Chemotherapy + photothermal therapy with hypoxia relief	Hypoxic solid-tumour model, in vivo	Catalase-driven O_2_ generation relieves hypoxia and overcomes related therapeutic resistance	[147]
Fe/Mn-doped ZIF-8-coated Bi_2_S_3_ loaded with methotrexate	Bi_2_S_3_: MRI (7 T) and X-ray CT, demonstrated in vivo	Photothermal + chemodynamic + chemotherapy	HepG2 hepatocellular carcinoma cells; tumour-bearing mice, in vivo	Synergistic PTT/CDT/chemo promoting cancer-cell apoptosis	[148]
Sr/Se co-doped ZIF-8 nanozyme	Primarily therapeutic	Chemotherapy + chemodynamic therapy (apoptosis + ferroptosis)	Tumour, in vivo	Synergistic regulated-cell-death antitumour effect	[149]
Hyaluronic-acid-coated CaO_2_/Ce6Cu-ZIF	Ce6 (fluorescence-capable)	Chemodynamic + photodynamic therapy with O_2_ self-supply	Tumour cells in vitro	CaO_2_-derived H_2_O_2_/O_2_ amplifies •OH and ^1^O_2_ generation; released Ca^2+^ additionally causes mitochondrial Ca^2+^ overload	[150]
Cell-membrane-coated Ce6/glucose-oxidase/heminZIF (mCGHZIF)	Ce6 (fluorescence-capable)	Starvation therapy + photodynamic therapy	4T1 breast cancer cells, in vitro	Glucose depletion with in situ O_2_ supply enhances PDT; homologous targeting and immune evasion	[151]
Folate-BSA-coated SeZIF-8 loaded with methotrexate	Primarily therapeutic (targeted delivery)	pH-responsive methotrexate chemotherapy	Breast cancer cells in vitro	Dual-targeted, acid-triggered methotrexate release	[152]
EGCGZIF-8	Primarily therapeutic	Photodynamic + chemodynamic antibacterial therapy	Infected wound, in vivo (mice)	ROS-based antibacterial action accelerates infected-wound healing	[153]

## 3. Conclusions and Outlook

Zeolite-based theranostics can be seen as the natural evolution of the applications reviewed here. In haemostasis, zeolites exploit adsorption and ion exchange to modulate a local biological process; in biomolecule separation and sensing, they act as biointerface materials for capture, stabilization, recognition, and signal generation; in theranostics, these functions converge in multifunctional platforms that integrate therapeutic delivery and diagnostic readout within a single material. Across all three areas, a consistent lesson emerges: the biomedical behaviour of zeolite-based materials is governed less by intracrystalline microporosity than by external surface area, hierarchical porosity, surface charge, and composite design—which is why nanosized and hierarchical architectures are central to the field’s future.

Realizing this potential will require systematic control of particle size, colloidal stability, protein-corona formation, biodegradation, biodistribution, and long-term safety, together with standardized protocols and validation in clinically relevant models. One requirement that cuts across all three areas, yet is reported only sporadically, is the chemical stability of the framework under the conditions of actual use. Alkaline elution buffers, physiological fluids, repeated regeneration cycles, and prolonged contact with tissue can each promote partial dissolution or dealumination. The consequences are a shift in surface charge, the release of silicate and aluminate species, and a loss of performance from one cycle to the next. Making post-use characterization routine—framework integrity, post-use Si/Al ratio, and dissolved silicon—would improve the comparability of published results and is arguably a prerequisite for moving any of these platforms beyond the laboratory. A candid reading of the present evidence base is equally necessary. Most of the recent theranostic literature that reaches in vivo validation concerns zeolite-like imidazolate frameworks rather than aluminosilicate zeolites, whose published record remains dominated by in vitro experiments and by biodistribution in healthy animals. It bears repeating that these two classes differ fundamentally in composition, degradation, and clearance (see Table 2): the ZIF results should be read as structural inspiration and proof of concept for stimuli-responsive design, not as direct evidence that non-biodegradable aluminosilicate zeolites will achieve comparable in vivo performance or clearance. For the moment, therefore, the theranostic promise of zeolites rests more on their physicochemical versatility than on demonstrated therapeutic outcomes, and the decisive step for the field will be a body of work in relevant disease models reporting efficacy, quantitative biodistribution, and clearance alongside the imaging readout. Addressed together, these challenges position zeolites not as passive carriers but as tunable inorganic scaffolds for next-generation image-guided and stimuli-responsive biomedical systems.

Looking ahead, we suggest a concrete and actionable agenda for the field. First, post-use framework characterization should become routine rather than exceptional: as set out in the reporting standard proposed in Section 2.3.3, every study that subjects a zeolite to biological fluids, alkaline elution, or regeneration cycles should document framework integrity by X-ray diffraction, the Si/Al ratio by ICP-OES and the dissolved silicon and aluminum species released, so that structural degradation and the attendant risk of cytotoxicity from leached ions can be assessed and compared across laboratories. Second, bio-interface interactions should be evaluated against standardized test matrices and protocols that specify ionic strength, protein conformational state, and desorption yield, resolving the present ambiguity between reversible and irreversible binding. Third, systemic nanozeolite studies should report dose-dependent accumulation, reticuloendothelial clearance, and long-term histology with the same rigour applied to efficacy.

Three emerging directions are likely to shape the next phase of the field. (i) Data-driven and machine-learning approaches to zeolite design, already transforming the discovery of new framework types and the prediction of adsorption behaviour, could be turned toward biomedical descriptors such as external-surface chemistry, protein-corona composition, and ion-release kinetics [154]. (ii) Hybrid inorganic–organic nanocarriers that graft the stimuli-responsive, degradable character of ZIFs onto the robust aluminosilicate framework could combine the stability of the former with the clearance profile of the latter. (iii) The deliberate engineering of controlled biodegradability into aluminosilicate nanozeolites—tuning dissolution rate through composition, Si/Al ratio, and surface passivation—would directly address the clearance limitation identified above and is perhaps the single most consequential materials challenge for the field. Pursued together, these directions would move zeolites from versatile but passive scaffolds toward rationally designed, safely clearable theranostic platforms.

## Figures and Tables

**Table 1:** Zeolite framework types referred to in this review, with their IZA three-letter codes, a representative material or mineral, the largest ring defining the pore aperture, the approximate aperture size, the dimensionality of the channel system and the context in which each framework appears in the following sections. Structural data follow the IZA-SC Database of Zeolite Structures [13]; MR = membered ring. Frameworks are grouped following the conventional classification of zeolites as small-, medium-, or large-pore according to the 8-, 10-, or 12-membered ring defining the channel opening.

Framework Type (Code)	Representative Material/Mineral	Largest Ring	Pore Aperture (Å)	Channel System	Relevance in This Review
LTA	Zeolite A (Linde 3A/4A/5A)	8-MR	4.1	3D	Haemostatic granules (Linde 5A); magnetic composites for nucleic acid extraction [21,22]
FAU	Zeolites X, Y and ultrastable Y (USY)	12-MR	7.4	3D	Protein adsorption and purification (USY); T_2_-MRI composites (NaY); bioimaging [23,24,25,26]
EMT	EMC-2 (hexagonal faujasite)	12-MR	6.9 × 7.3	3D	Plasma protein-corona studies on nanosized zeolites [27]
MFI	ZSM-5, silicalite-1	10-MR	5.1 × 5.5	3D	pH-responsive doxorubicin delivery (ZSM-5/chitosan nanodisks) [28]
BEA	Zeolite beta (Na-BEA)	12-MR	6.6 × 6.7	3D	pH-selective protein fractionation; protein refolding supports [29,30]
CHA	Chabazite	8-MR	3.8	3D	Mesoporous chabazite grown on cotton for flexible haemostatic dressings [31]
FER	Ferrierite (K-FER)	10-MR	4.2 × 5.4	2D	Strong, poorly reversible protein binding at high SiO_2_/Al_2_O_3_ [29,32]
LTL	Zeolite L	12-MR	7.1	1D	Site-selective biomolecule immobilization; Gd/^89^Zr multimodal imaging [33,34]
HEU	Heulandite/clinoptilolite	10-MR	3.0 × 7.6	2D	Most studied natural zeolite for in vivo safety; magnetic theranostic particles [35,36]

## Data Availability

No new data were created or analyzed in this study. Data sharing is not applicable to this article.

## Abbreviations

The following abbreviations are used in this manuscript:

ACS	Advanced Clotting Sponge
ACS+	Advanced Clotting Sponge Plus
AIPO	aluminophosphate (AIPO)
APTT	activated partial thromboplastin time
BEA	zeolite beta framework type
BSA	bovine serum albumin
CaCu-ZG	calcium–copper zeolite gauze
CDT	chemodynamic therapy
Ce6	chlorin e6
CG	Combat Gauze
CHA	chabazite framework type
CT	computed tomography
DNA	deoxyribonucleic acid
DOX	doxorubicin
EGCG	epigallocatechin gallate
EMT	hexagonal faujasite framework type
ERI	erionite framework type
FAU	faujasite framework type
FDA	US Food and Drug Administration
FER	ferrierite framework type
HEU	heulandite/clinoptilolite framework type
IARC	International Agency for Research on Cancer
ICG	indocyanine green
ICP-OES	inductively coupled plasma optical emission spectrometry
IZA	International Zeolite Association
IZA-SC	International Zeolite Association Structure Commission
LC-MS/MS	liquid chromatography–tandem mass spectrometry
LTA	Linde Type A framework type
LTL	zeolite L framework type
MFI	ZSM-5/silicalite-1 framework type
MOF	metal–organic framework
MRI	magnetic resonance imaging
MSN	mesoporous silica nanoparticle
MWCNT	multi-walled carbon nanotube
NIR	near-infrared
PDT	photodynamic therapy
PEG	poly(ethylene glycol)
PET	positron emission tomography
pI	isoelectric point
PT	prothrombin time
PTT	photothermal therapy
PVA	poly(vinyl alcohol)
QC	QuikClot
RES	reticuloendothelial system
RNA	ribonucleic acid
ROS	reactive oxygen species
RSV	respiratory syncytial virus
SAPO	silicoaluminophosphate
SBU	secondary building unit
scFv	single-chain variable fragment
SDT	sonodynamic therapy
STI	stilbite framework type
TME	tumour microenvironment
USY	ultrastable zeolite Y
Z-RCA	zeolite–regenerated cellulose aerogel
ZIF	zeolitic imidazolate framework
ZSM-5	Zeolite Socony Mobil-5
